# Oocyte quality assessment in marine invertebrates: a novel approach by fluorescence spectroscopy

**DOI:** 10.1186/s40659-022-00403-4

**Published:** 2022-11-12

**Authors:** Alessandra Gallo, Maria Consiglia Esposito, Raffaele Boni, Elisabetta Tosti

**Affiliations:** 1 Department of Biology and Evolution of Marine Organisms, Stazione Zoologica Anton Dohrn, Naples, NA Italy; 2 Department of Sciences, University of Basilicata, Potenza, PZ Italy

**Keywords:** Egg, Fluorescence spectroscopy, Intracellular pH, Intracellular ROS level, Mitochondrial activity, Mussel, Oocyte quality, Plasma membrane lipid peroxidation, Sea urchin, Viability

## Abstract

**Background:**

The assessment of oocyte quality is, nowadays, a major challenge in aquaculture, oocyte cryopreservation, and environmental science. Oocyte quality is a determining factor in fertilization and embryo development; however, there is still a lack of rapid and sensitive cellular markers for its assessment. Currently, its estimation is predominantly based on morphological analysis, which is subjective and does not consistently reflect the developmental competence of the oocytes. Despite several recent studies investigating molecular markers related to oocyte quality, methods currently available for their determination pose various technical challenges and limitations. In this study, we developed a novel approach based on fluorescence spectroscopy to assess different intrinsic physiological parameters that can be employed to evaluate egg quality in marine invertebrates that are widely used as animal models such as sea urchins and mussels.

**Results:**

Different physiological parameters, such as viability, mitochondrial activity, intracellular ROS levels, plasma membrane lipid peroxidation, and intracellular pH, for egg quality evaluation have been successfully assessed in sea urchins and mussels by using specific fluorescent dyes and detecting the fluorescent signals in eggs through fluorescence spectroscopy.

**Conclusions:**

Based on our findings, we propose these physiological markers as useful predictors of egg quality in marine invertebrates; they can be estimated rapidly, selectively, and sensitively by employing this novel approach, which, due to the speed of analysis, the low cost, and easy use can be considered a powerful analytical tool for the egg quality assessment.

## Background

Oocytes are highly specialized cells that undergo the unique processes of meiosis and fertilization and execute a molecular program for development [1].

Oocyte quality has been defined as the ability of oocytes to be fertilized and produce viable progeny. In marine species, the evaluation of oocyte quality is commonly based on morphological criteria, such as size, shape, transparency, chorion aspects, distribution and volume of lipid droplets, lipid content and floatability rate, as well as fertilization and developmental success assessment [2, 3]. However, the morphological classification of oocytes is subjective and does not consistently reflect their ability to grow and develop [4]. Moreover, the assessment of fertilization and developmental success is time-consuming and, depending on the species, technically difficult to be estimated [2]. Hence, to date, an effective and simple proxy of oocyte quality parameters has not been established yet making difficult for an accurate assessment of the female gamete quality before fertilization. To overcome this issue, different predictive markers of oocyte quality have been proposed [5, 6]. Many investigations have been designed to gain new insights into the mechanisms underlying oocyte quality based on a differential analysis applied to molecular, biochemical, transcriptomic, or proteomic data. Biochemical content determinations have been exploited for oocyte quality evaluation [7–9], and, recently, the oocyte proteomic profile has been proposed as quality estimator [10, 11]. Nevertheless, the complexity of the techniques applied to assess gene expression and protein profile is a major bottleneck for the use of molecular markers in the oocyte quality assessment [12].

Several physiological parameters, such as viability, mitochondrial activity, and reactive oxygen species (ROS) levels have been also reported as useful markers of oocyte quality in marine invertebrates.

Viability is surely a fundamental requirement of oocyte quality; however, it is not per se sufficient to assure oocyte developmental competence [13].

On the other side, mitochondrial functionality is one of the main used indicators for estimating oocyte quality [14]. In the oocyte, mitochondria play a central role in cellular metabolism providing energy for successful cytoplasm and nucleus maturation, fertilization, embryo development, calcium homeostasis, and apoptosis initiation. They are synthesized and accumulated in high numbers during oogenesis so that mature oocytes contain thousands of these organelles localized according to a gradient, which, after fertilization, are distributed into the daughter cells supporting the development up to the larval stage [15–17]. Mitochondria number, distribution, and activity are narrowly tied with oocyte quality [18, 19]. High-polarized mitochondria have been associated with many metabolic activities including ATP production and calcium homeostasis as well as with fertilization and developmental competence [20]. On the other hand, low-polarized mitochondria have been correlated to low ATP production and low levels of metabolic activity in meiotic arrest oocytes, which are in a quiescent state [21], but also to oocyte maturation arrest, fertilization, and embryo development failures. In light of this, mitochondria activity is considered a determinant factor of oocyte quality [22]; indeed any stressor affecting mitochondrial functionality might impair the oocyte competence.

In the oocyte, the production of ROS occurs during the metabolic processes at a controlled rate. ROS act as signalling molecules in oocyte physiology regulating nuclear maturation, meiotic arrest as well as resumption, fertilization, and embryo development [23]. The generation and the clearance of ROS are involved in the maintenance of oocyte quality and thereby, female reproductive health. Several studies have drawn a compelling link between oxidative stress (OS) and the decline in oocyte quality. Indeed, increased levels of ROS beyond the physiological range may induce OS, which deteriorates oocyte quality by arresting the cell cycle and inducing apoptosis [24, 25].

In marine invertebrates, the aforementioned oocyte quality parameters have been determined using fluorescent dyes combined with the epifluorescence microscopy [26–28] or flow cytometry [29, 30]. The former has the advantage of identifying the fluorescent signals in individual cells; nevertheless, only few cells of the same sample can be evaluated. The latter allows for the assessment of several parameters on small samples in a very short time providing rapid and accurate results. However, the flow cytometry is an expensive technique that also requires skilled and highly trained operators to perform it. Thereby, there is an urgent need to develop new approaches allowing the rapid assessment of oocyte quality by employing simple, economic, and feasible protocols. To address this issue, this study developed a novel approach based on fluorescence spectroscopy to evaluate different physiological parameters related to oocyte quality, such as viability, mitochondrial activity, intracellular ROS levels, plasma membrane lipid peroxidation (LPO), and the intracellular pH (pH_i_) in marine invertebrates. To date, a similar approach has been well‐developed to estimate several quality parameters in marine invertebrate spermatozoa; however, up to date, it has not been applied to oocytes.

Besides viability, mitochondrial activity, and intracellular ROS levels, in this study, new potential markers of oocyte quality in marine invertebrates, such as LPO and the pH_i_, have been investigated. The regulation of pH_i_ is a fundamental homeostatic process that is essential for oocyte function and viability. Small pH_i_ shifts are involved in the critical transitions of the oocyte status, as during meiotic maturation and following oocyte activation and fertilization [31, 32], and trigger a cascade of cellular events that initiate embryo development [33]. On the other hand, major alterations in pH_i_ have been demonstrated to affect cellular events regulating embryo development in sea urchins [34–36].

It is well known that ROS affect gamete integrity also through LPO [37, 38], which is an oxidative process that modifies the structure and the properties of plasma membrane lipids affecting the oocyte quality [39].

Overall, the objective of this study is to develop a rapid and sensitive approach to assess different physiological parameters that can be used as markers of the egg quality in sea urchins and mussels. These play important ecological roles in marine ecosystems, but also hold high commercial interest and are widely employed in biomonitoring programs and ecotoxicological studies; thereby, they are among the most harvested species. Oocyte cryopreservation may provide a great contribution to their availability increasing the accessibility of gamete supplies out-of-season and enhance efficiency in selective breeding. These may allow access to genetic resources all the year regardless of the seasonal reproductive cycle for fundamental research, ecotoxicology studies, aquaculture and conservation.

## Results

### Confocal laser scanning microscopic analysis

**Fig. 1:**
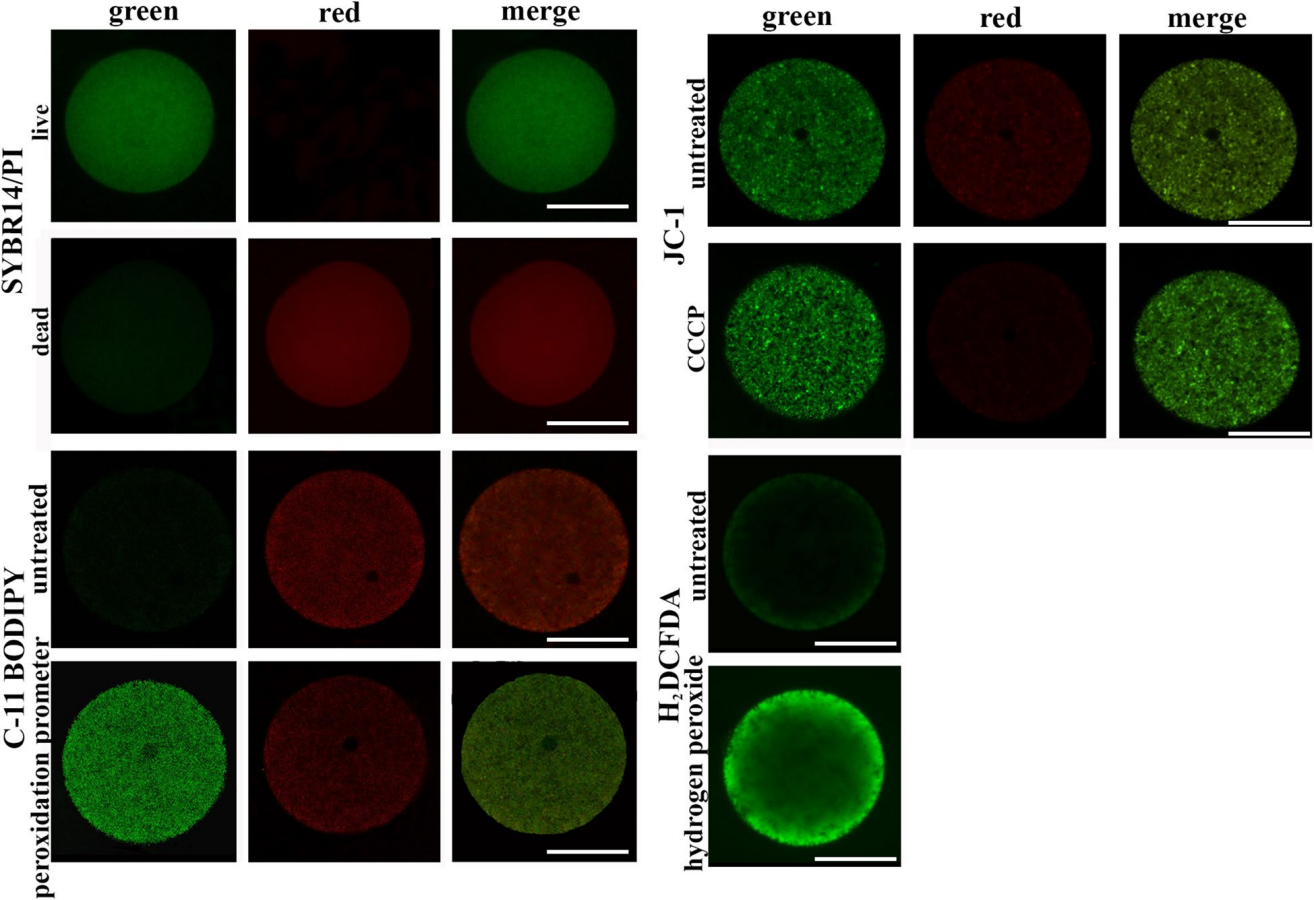
Representative fluorescence images of sea urchin eggs stained with: SYBR-14/Propidium iodide (PI) for viability assessment; JC-1 for mitochondrial membrane potential determination; H_2_DCFDA for intracellular ROS level evaluation; and C11-BODIPY^581/591^ for plasma membrane lipid peroxidation estimation. For each fluorochrome, eggs have been divided in untreated (first row) and treated (second row) with: glutaraldehyde for SYBR14/PI; peroxidation promoters (ferrous sulfate and vitamin C) for C11-BODIPY^581/591^; hydrogen peroxide for intracellular ROS; carbonyl cyanide m-chlorophenyl hydrazone (CCCP) for JC-1 (positive controls). For all dyes, the merge image includes the green and the red channels, except for H_2_DCFDA dye, for which only the green channel has been showed. Scale bars 50 μm

Before the spectrofluorometric analysis, all the fluorochromes employed in this study have been evaluated by confocal laser scanning microscopy. The microscopic examination revealed their localization within the specific egg compartment in all analysed species (Fig. 1).

### Viability

**Fig. 2:**
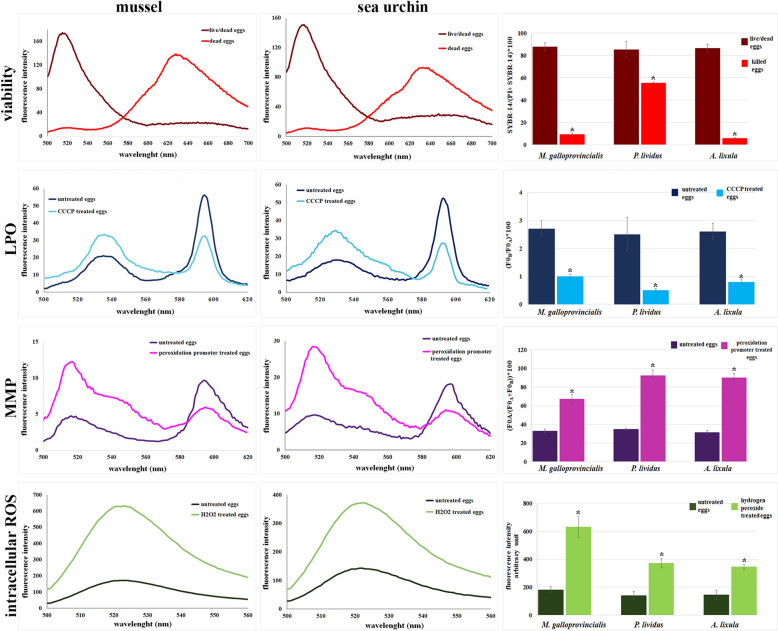
Egg quality assessment in mussel and sea urchin. In the left and middle columns, there are representative fluorescence emission spectra recorded by spectrofluorimetry in the *Mytilus galloprovincialis* and *Paracentrotus lividus* eggs stained with: SYBR-14/Propidium iodide (PI) for viability assessment; JC-1 for mitochondrial membrane potential (MMP) determination; H_2_DCFDA and hydrogen peroxide for intracellular ROS level evaluation; and C11 BODIPY.^581/591^ for plasma membrane lipid peroxidation (LPO) estimation. For each fluorochrome, eggs have been divided in untreated and treated with: glutaraldehyde for SYBR14/PI; peroxidation promoters (ferrous sulfate and vitamin C) for C11-BODIPY581/591; hydrogen peroxide for intracellular ROS; carbonyl cyanide m-chlorophenyl hydrazone (CCCP) for JC-1 (positive controls). In the right column, mean values (± SD) of viability, MMP, intracellular ROS levels, and LPO recorded in untreated and treated eggs of the mussel (*M. galloprovincialis*) and the sea urchin (*P. lividus* and *A. lixula*) were compared. * indicates statistically significant differences (P ≤ 0.05)

The egg viability has been evaluated by the SYBR-14/PI fluorescence intensity ratio and the results are reported in Table 1. Egg death induction treatment induced a significant increase in PI and a reduction in SYBR14 fluorescence intensities in all analysed species. This resulted in a significant reduction in the estimated viability value (88.0 ± 3.6 *vs.* 9.5 ± 1.0 in *M. galloprovincialis*, 85.2 ± 7.5 *vs.* 5.5 ± 0.8 FI in *P. lividus*, and 86.5 ± 3.5 *vs.* 5.9 ± 0.4 FI in *A. lixula:* P < 0.01) (Fig. 2).

**Table 1:** Viability, MMP, ROS, LPO, and pH_i_ values recorded in mussel and sea urchin eggs

		Mussel	Sea urchin	
		*Mytilus galloprovincialis*	*Paracentrotus lividus*	*Arbacia lixula*
Viability	SYBR-14/(PI + SYBR-14)*100	88.0 ± 3.6	85.2 ± 7.5	86.5 ± 3.5
MMP	(F0_B_/F0_A_)*100	2.7 ± 0.3	2.5 ± 0.6	2.6 ± 0.3
ROS	arbitrary unit	181.9 ± 24.5	142.1 ± 28.3	146.8 ± 34.7
LPO	(F0_A_/(F0_A_ + F0_B_))*100	32.9 ± 2.1	34.8 ± 1.2	31.5 ± 2.2
pH_i_		8.3 ± 0.11	7.9 ± 0.14	7.80 ± 0.08

Mean values (± SD) of the viability, *MMP* mitochondrial membrane potential, intracellular ROS levels, plasma membrane lipid peroxidation (LPO), and *pH*_*i*_ intracellular pH measured in mussel and sea urchin eggs by employing specific fluorochrome for each parameter coupled with fluorescence spectroscopy analysis

### Mitochondrial membrane potential (MMP)

The MMP has been calculated as a ratio of the red (J-aggregates) and green fluorescence (J-monomers) reflecting high and low mitochondrial potential, respectively. In the eggs of all analysed species, low MMP values have been measured (Table 1). The treatment with the mitochondrial uncoupler carbonyl cyanide m-chlorophenyl hydrazone (CCCP) resulted in a significant decrease in JC-1 fluorescence ratio indicating the MMP collapse: 2.7 ± 0.3 *vs.* 1.0 ± 0.08 a.u. in *M. galloprovincialis*, 2.5 ± 0.6 *vs.* 0.5 ± 0.06 a.u. in *P. lividus*, and 2.6 ± 0.3 *vs.* 0.8 ± 0.09 a.u. in *A. lixula* (P < 0.05) (Fig. 2).

### Intracellular ROS levels

The oocyte ROS production has been detected by using the probe H_2_DCFDA, whose fluorescence signal upon oxidation by hydrogen peroxide allows the quantification of ROS levels (Table 1). After egg exposure to H_2_O_2_, a significant increase in the intracellular ROS levels has been recorded in all analysed species (181.9 ± 24.5 *vs.* 632.5 ± 77.91 in *M. galloprovincialis*; 142.1 ± 28.3 *vs.* 372.4 ± 34.7 in *P. lividus;* and 146.8 ± 34.7 *vs.* 346.0 ± 17.44 in *A. lixula;* P < 0.05) (Fig. 2).

### Plasma membrane lipid peroxidation

The LPO has been estimated by using the fluorochrome C11 BODIPY^581/591^, which shifts its fluorescence from red to green upon oxidation. The ratio between red and green fluorescence provides a measure of LPO in the eggs of all analysed species (Table 1). After the egg treatment with peroxidation promoters, the emission peak at 595 nm (red fluorescence) decreased whereas that at 520 nm increased; consequently, the ratio significantly increased (32.9 ± 2.1 *vs.* 67.3 ± 4.8 FI in *M. galloprovincialis*; 34.8 ± 1.2 *vs.* 92.4 ± 5.9 in *P. lividus;* and 31.5 ± 2.2 *vs.* 90.2 ± 4.8 in *A. lixula*; P < 0.05) (Fig. 2).

### Intracellular pH

Table 1 showed the average values of the pH_i_ measured in the mussel and sea urchin eggs by employing the fluorochrome 2′-7′-bis (carboxyethyl)-5(6)-carboxyfluorescein (BCECF) and determining the pH-dependent ratio of emission fluorescence intensity at 535 nm when the dye is excited at 490 and 440 nm.

## Discussion

The assessment of gamete quality in marine species has become an important issue in aquaculture and for the development of cryopreservation protocols as well as environmental science. However, the egg quality evaluation is more challenging since, to date, the assessment methods are very limited. In this study, we developed a novel approach using fluorescence staining in combination with spectrofluorimetric analysis for the assessment of egg quality in marine invertebrates. Nowadays, this method is commonly used to evaluate sperm quality in different marine species [40–44]; whereas, this study represents the first report on the development and validation of this approach for the evaluation of different egg quality parameters, such as viability, mitochondrial membrane potential, intracellular ROS level, plasma membrane lipid peroxidation, and the pH_i_ in mussels and sea urchins.

Herein, the egg viability has been estimated using the dual DNA staining, employing the fluorochrome SYBR-14 and PI. We demonstrated that this staining results effectively in assessing viability in mussel and sea urchin eggs as confirmed by the positive control. Previously, this dual fluorescent staining was successfully employed to evaluate oocyte viability only in the Pacific oyster *Crassostrea gigas* [29].

The MMP is a key indicator of mitochondrial activity because it correlates to cell capacity to generate ATP by oxidative phosphorylation. Several fluorescent lipophilic cationic dyes are used for measuring MMP, such as TMRM (tetramethylrhodamine methyl ester), TMRE (tetramethylrhodamine ethyl ester), Rhodamine 123, and JC-1. Among these, JC-1 has been demonstrated the most accurate and specific probe to estimate MMP [45, 46]. To date, JC-1 has been used to assess mitochondrial activity in the oocyte of a few marine invertebrates species such as the ascidian *Styela plicata* [19] and the corals *Junceella sp* [47]*.* In this study, we validated the use of JC-1 for measuring MMP changes in the eggs of sea urchins and mussels as confirmed by CCCP treatment. Indeed, in CCCP treated eggs, we observed a reduction of the red-to-green fluorescence intensities ratio, which indicates both the inhibition of JC-1 aggregate formation due to the MMP decrease and the exclusive localization of JC-1 in the mitochondria. The low MMP values detected in the oocytes of the three analyzed species may be due to the presence in the eggs of mitochondria, which are more transcriptionally and bioenergetically silent.

The intracellular ROS levels are considered a reliable predictor of oocyte developmental competence. To date, the best approach employed to detect ROS production is a fluorescence-based system because of its high sensitivity. The 2′,7′-dichlorodihydrofluorescein diacetate (DCFH-DA)-based staining is the most commonly used fluorochrome for detecting intracellular ROS in the oocytes of different species [48–51]. In marine invertebrates, it has been applied for ROS detection only in oyster oocytes, and, to the best of our knowledge, currently, no literature is available on the assessment of intracellular ROS in oocytes of different marine invertebrate species. In this study, we demonstrated that DCFH-DA staining could be effectively used to measure intracellular ROS levels in mussel and sea urchin eggs. The efficacy of this method has been supported by using positive controls in which the eggs were exposed to an oxidative stimulus that caused a large increase in fluorescent intensity values.

ROS may affect egg quality primarily through LPO. Nowadays, different biochemical assays for LPO assessment have been developed; however, they are relatively insensitive and require a large number of cells. Recently, a new approach for evaluating LPO has been developed, involving the fluorochrome C11 BODIPY^581/591^. This probe has been demonstrated to be very suitable for sensitive quantitation of LPO providing a simple ratiometric method for detecting the oxidative degradation of cellular lipids. The ratio of red to green fluorescence provides a measure of lipid peroxidation, which is independent of factors such as lipid density that may influence measurement with single emission probes. Previous studies indicated that C11-BODIPY^581/591^ is a very effective probe for monitoring LPO in spermatozoa [52–54]; however, to our knowledge, it has never been used to assess LPO in eggs. In this study, we evaluated for the first time the applicability of this probe for the LPO assessment in marine invertebrate eggs. The obtained results indicate that C11-BODIPY^581/591^ is a valuable tool to quantify LPO in mussel and sea urchin eggs.

The pH_i_ is an important physiological determinant of egg quality since it plays important roles in different processes, such as protein synthesis, oocyte activation, and cytoskeletal rearrangement [55, 56]. Different approaches employed to determine pH_i_ include the use of pH-sensitive fluorescent dyes. The fluorescence assessment of pH_i_ was commonly performed by using the pH indicator BCECF, which is considered suitable for measuring pH_i_ since it is well retained in the cell and is not susceptible to leakage [57]. Up to date, BCECF has been employed to measure the pH_i_ in oocytes and eggs of marine invertebrates such as surf clam [58], starfish [59], sea urchin [60], and polychaete [61]. Nevertheless, it has not been yet validated for pH_i_ assessment in the eggs of the sea urchin *P. lividus* and *A. lixula*. Furthermore, to the best of our knowledge, no report is available on the assessment of pH_i_ in mussel eggs based on BCECF staining. In this study, we demonstrated that BCECF is effective in assessing pH_i_ in mussel eggs and confirmed that it is applicable to pH_i_ measurements of sea urchin eggs.

Up to date, the fluorescent dye accumulation in eggs has been detected by flow cytometry and fluorescence microscopy, which present different disadvantages. In this study, we have evaluated the suitability of fluorescence spectroscopy to analyse eggs labelled with a specific fluorophore demonstrating that this technique allows sensitive and accurate detection of the signal in stained eggs. Furthermore, the use of a microplate reader has several advantages. In fact, the microplate-based method allows the simultaneous analysis of the different egg physiological parameters within a few minutes; moreover, it requires a small sample volume (200 µL), which minimized the expenses required for the testing procedures, in particular those of the fluorophore used.

Based on our findings, we have validated different physiological parameters such as viability, mitochondrial activity, intracellular ROS levels, LPO, and pH_i_ as useful indicators of healthy eggs in marine invertebrates; nevertheless, further investigations are still necessary to evaluate if these parameters are also predictive biomarker of a high developmental competence. Moreover, we demonstrated that, due to the speed of analysis, inexpensive, and easy use, combining fluorescent staining and fluorescence spectroscopy analysis is a powerful analytical tool for egg quality assessment allowing the rapid, selective, and sensitive measurements of different egg quality parameters in marine invertebrates.

## Conclusions

In the present study, a novel approach has been developed for evaluating several physiological parameters in mussel and sea urchin eggs based on fluorescence staining and fluorescence spectroscopy. The developed method is easy-to-perform and allows a rapid and sensitive assessment of the viability, mitochondrial activity, intracellular ROS, LPO, and pH_i_ in eggs of marine invertebrates providing markers of egg quality and, hence, an assessment of the reproductive potential of the eggs. This approach represents a suitable alternative to the methods used so far for the analysis of the eggs with fluorescent dyes and has some advantages over these as it is less time-consuming and allows the evaluation of a large number of cells. Finally, it may be a promising and valuable tool to investigate the toxicity and mechanism of action of environmental pollutants in the framework of reproductive risk assessment.

## Methods

### Animal collection and spawning

#### Mussel

Adult individuals of *Mytilus galloprovincialis* were collected from an aquaculture farm situated on the Bay of Naples (Italy) and transported in a cool box to the Marine Biological Resources service of Stazione Zoologica Anton Dohrn. Here, mussels were acclimated in glass tanks with running natural seawater (NSW, 38 g/L salinity, pH 8.2 ± 0.1, 18 °C) for at least 3 days until the experiments. Mussels were induced to spawn by thermal shock moving the animals from a tank containing seawater at 18 °C to one that contains seawater at 24 °C. Emitted eggs were collected in glass beakers and filtered with a 70 μm sieve. Finally, eggs were diluted to the desired concentration in filtered (Millipore 0.22 mm; MilliQ, Medford, MA) natural seawater (FNSW).

#### Sea urchin

Adult sea urchins *Paracentrotus lividus* and *Arbacia lixula* were collected from the Gulf of Naples. After collection, the animals were acclimated in glass tanks filled with circulating natural seawater (NSW, 38 g/L salinity, pH 8.2 ± 0.1, 18 °C) for at least seven days until use. Eggs were obtained from mature animals by injecting 0.5 M KCl solution into the coelom. After injection, females were inverted over a beaker containing FNSW and left to release eggs. Egg morphology and concentration were evaluated by a light microscope using a counting chamber. Finally, eggs were diluted to the desired concentration in FNSW.

### Egg quality parameters

#### Viability

Egg viability was estimated by employing the fluorochrome SYBR-14 in combination with propidium iodide (PI). The former penetrates living eggs emitting a bright green fluorescence, whereas PI penetrates only cells that have lost membrane integrity, and emits in the red fluorescence range. Aliquots of egg suspension (1000 oocytes/mL) were stained by adding 100 µM SYBR-14 and incubating for 15 min in the dark at 18 ± 1 °C. After that, 12 μM PI was added and samples were further incubated for 15 min at 18 ± 1 °C in the dark. To validate the SYBR-14 and PI staining, eggs were killed by incubating for 1 h in glutaraldehyde 4% in FNSW. Finally, SYBR 14 and PI fluorescence intensity peaks were measured with the microplate reader setting the excitation wavelengths at 488 nm and at 545 nm recording the emission spectra in the range of 500–560 nm and 570–700 nm, respectively.

### Mitochondrial membrane potential *(MMP)*

MMP was measured using the potential-dependent fluorochrome 5,5′,6,6′-tetrachloro-1,1′,3,3′-tetraethylbenzimidazolylcarbocyanine iodide dye (JC-1; Life Technologies, Milan, Italy). JC-1 enters selectively into mitochondria where it can exist in two forms, monomeric or aggregate, depending upon mitochondrial membrane potential. The monomeric form predominates in mitochondria with low membrane potential and emits in the green wavelength (525–530 nm), whereas the aggregate form accumulates in mitochondria with high MMP and emits in the orange/red wavelength (~ 595 nm). The JC-1 aggregate/monomer ratio is assumed to be proportional to MMP. Aliquots of 1000 eggs/mL were incubated with 5 μM JC-1 for 30 min in the dark at 18 °C, then were washed, re-suspended in FNSW, and analysed to the microplate reader. The fluorescence spectra were recorded by setting the excitation wavelength at 488 nm and the emission spectrum in the range of 500–650 nm. The MMP was calculated as a ratio of the fluorescence peak values at ~ 595 nm and ~ 530 nm. Positive control samples were prepared by incubating JC-1-loaded eggs with 5 µM CCCP.

#### Intracellular ROS levels

The intracellular ROS levels were determined by using the 2′,7′-dichlorodihydrofluorescein diacetate (H_2_DCFDA, Life technologies, ThermoFisher Scientific, Milan, Italy). This is a membrane-permeable, non-fluorescent dye that, inside cells, is hydrolysed by intracellular esterase into DCFH, which can be oxidized by the intracellular hydrogen peroxide (H_2_O_2_) to the fluorescent compound DCF, whose fluorescence is proportional to ROS production. Aliquots of 1000 eggs/mL were incubated with 10 μM DCFH-DA for 30 min in the dark at 18 °C. After that, samples were washed and further incubated for 30 min in FNSW. After incubation, eggs were washed, suspended in FNSW, and transferred to a 96-well plate for spectrofluorometric analysis. The intracellular H_2_O_2_ levels were evaluated in arbitrary units (a.u.) on peak fluorescence intensity of emission spectra recorded from 500 to 560 nm wavelengths (peak at ~ 525 nm) setting the excitation wavelength at 488 nm. Positive control was prepared by incubating aliquots of egg suspension with 2.5 µM hydrogen peroxide.

#### Plasma membrane lipid peroxidation (LPO)

LPO of the egg plasma membrane was quantified using the fluorescent probe C11-BODIPY^581/591^ (4,4-difluoro-5-(4-phenyl-1,3-butadienyl)-4-bora-3a,4a-diaza-s-indacene-3-undecanoic acid; Life Technology, Milan, Italy). This is a lipophilic probe and a fatty acid analogue, which is easily incorporated into membranes and is sensitive to fatty acid oxidation changing from red (590 nm) to green (520 nm) fluorescence upon oxidation. Aliquots of eggs (1000 eggs/mL) were incubated with 5 µM C11-BODIPY^581/591^ in DMSO for 30 min in the dark at 18 °C. Then, eggs were washed and suspended in FNSW. Positive control was prepared by incubating egg aliquots with two peroxidation promoters (150 µM ferrous sulfate and 750 µM vitamin C). The spectrofluorometric analysis was performed recording the fluorescence intensity spectra at 500–650 nm emission wavelengths, after excitation at 488 nm. Lipid peroxidation value was determined as a ratio between fluorescence emission peak value at  ~ 520 nm and the sum of fluorescence emission peak values at  ~ 520 and  ~ 590 nm.

#### Intracellular pH (pH_i_)

The pH_i_ was evaluated using the pH-sensitive fluorescent dye 2′,7′-bis-(2-carboxyethyl)-5-(and-6) carboxyfluorescein acetoxymethyl ester (BCECF-AM; Life Technology, Milan, Italy). This dye is able to freely diffuse through the plasma membrane and, in the cell, it is hydrolyzed by esterases resulting in the cytoplasmic indicator BCECF whose fluorescence intensity depends on the pH_i_. The ester form of BCECF (5 µM BCECF-AM) was loaded to the egg suspensions (1000 cell/mL) and incubated in the dark at 18 °C for 30 min. Then, eggs were washed, resuspended in FNSW, and transferred to a 96-well plate for spectrofluorometric analysis. The pH_i_ value was measured by alternately exciting the samples at 440 nm and 490 nm and calculating the ratio of the fluorescence emission peak values at 535 nm. This ratio was converted in pH_i_ by using a calibration curve, which was constructed on each experiment by incubating aliquots of the eggs in a calibration buffer solution (135 mM KCl, 5 mM HEPES, 290 mOsm) set at pH 6.5, 7.2, and 8.0, in presence of 5 µM nigericin (Sigma Aldrich, Milan, Italy). Nigericin equilibrates intracellular and extracellular pH acting as an antiporter, promoting K^+^/H^+^ exchange. As consequence, the fluorescence intensity is linearly associated with the internal/external pH. By using the calibration curve equation, the ratios between the emission peak values obtained following excitation at 440 and 490 nm wavelength were transformed into pH_i_ values.

### Confocal laser scanning microscopy

Confocal microscopy (Zeiss LSM 510) was employed for each dye in loaded eggs mounted on a microscope slide to assess the correct fluorophore localization within a specific cell compartment.

### Spectrofluorometric analysis

The fluorescence of eggs was quantified on the microplate reader (Tecan Infinite^®^ m1000 pro). For each parameter, three replicate measurements were made by transferring aliquots of loaded eggs into three wells of a black polystyrene 96-well plate with a clear bottom. The plate reader is set to fluorescence-intensity mode to record the emission spectrum of each fluorochrome.

### Statistical analysis

Ten biological replicates have been performed for each egg physiological parameter and data are presented as the mean ± standard deviation (SD). The one-way analysis of variance followed by Fisher’s least significant difference test has been performed to test for significant differences between untreated and treated eggs. The significance level was set at P = 0.05.

## Data Availability

The datasets used and/or analyzed during the current study are available from the corresponding author on reasonable request.
